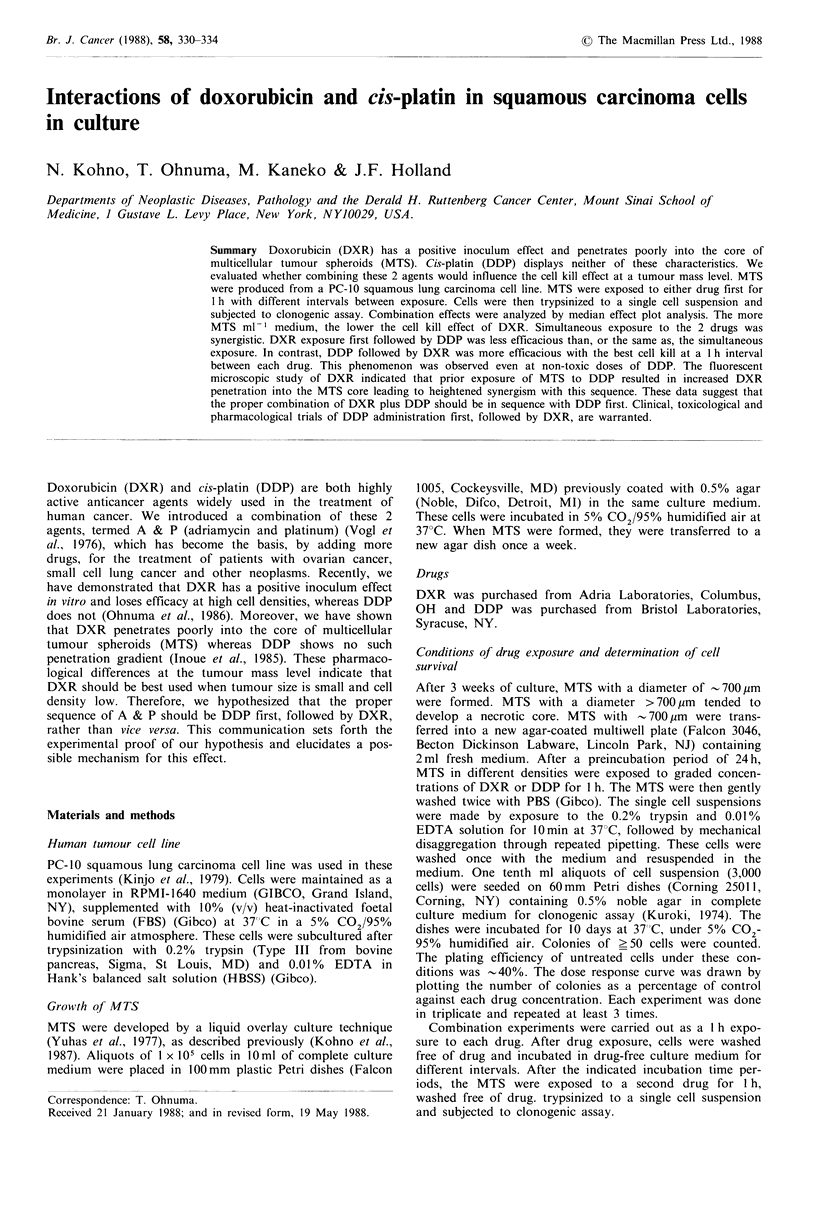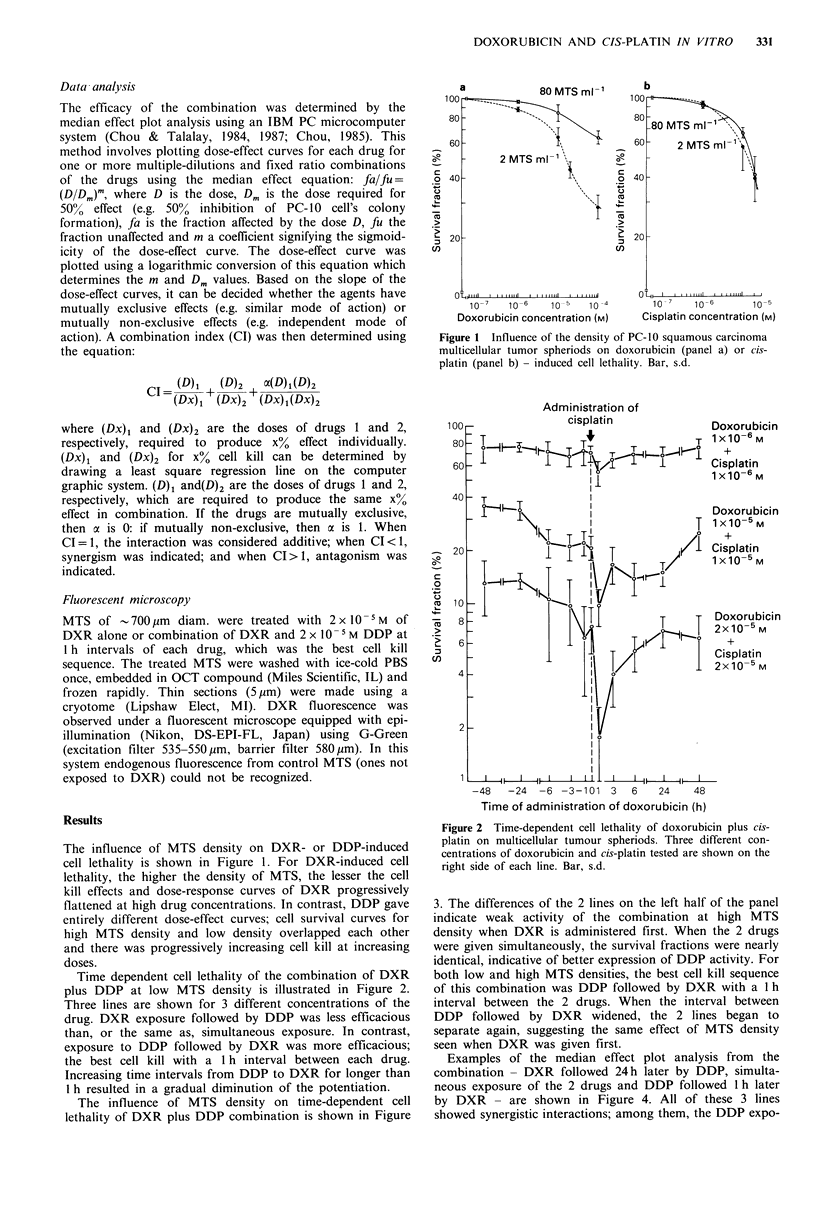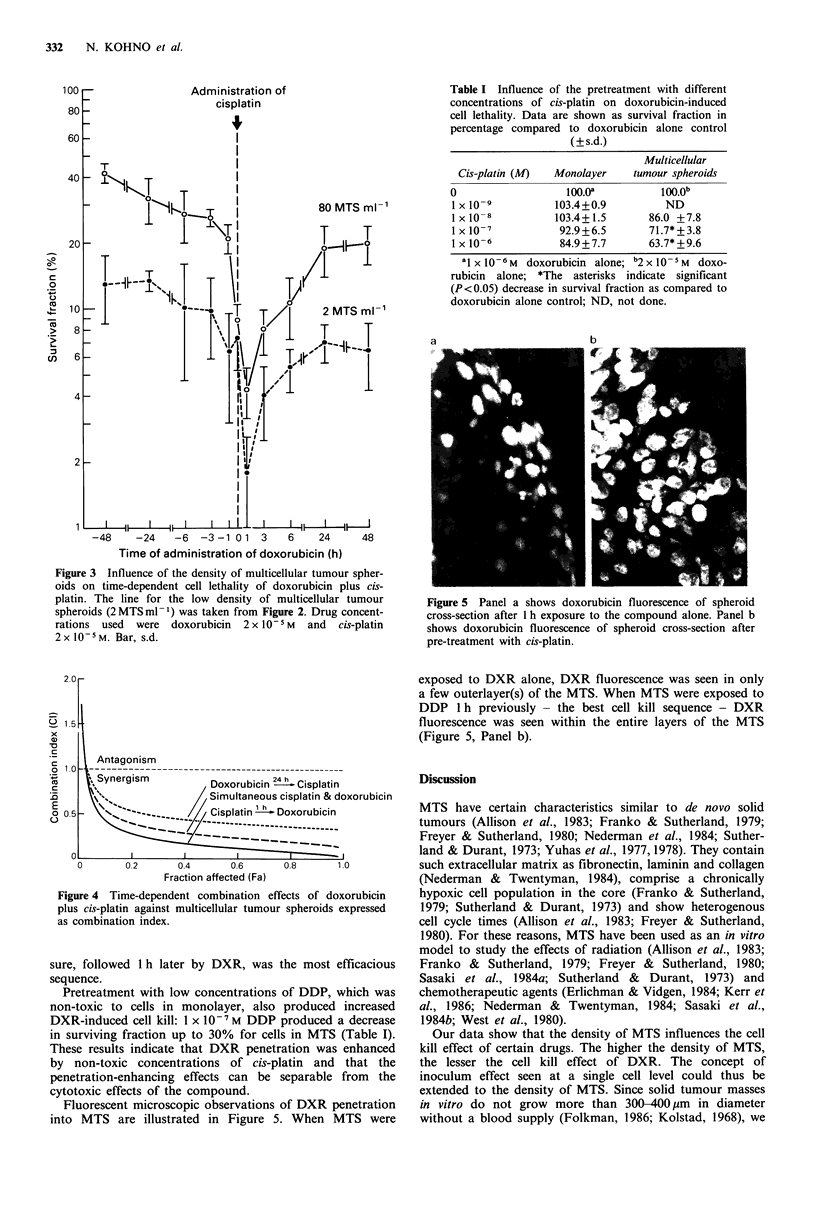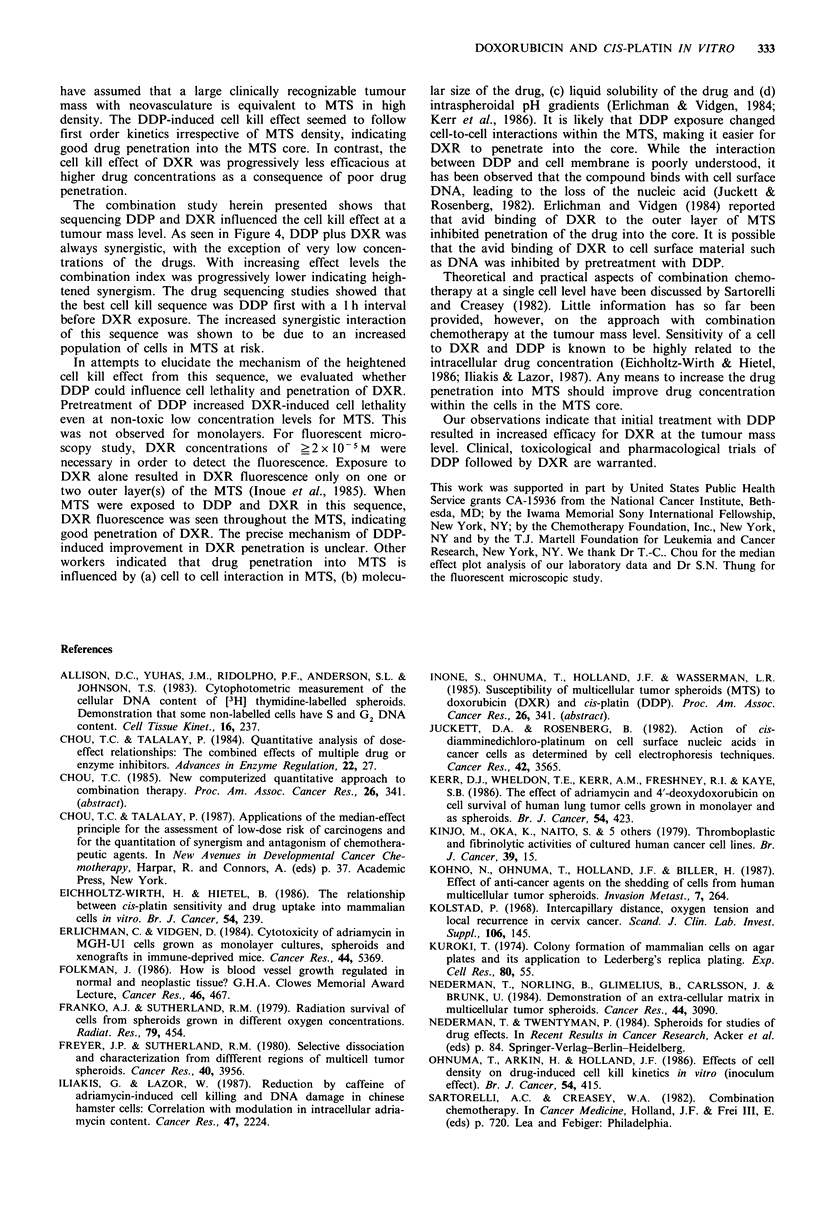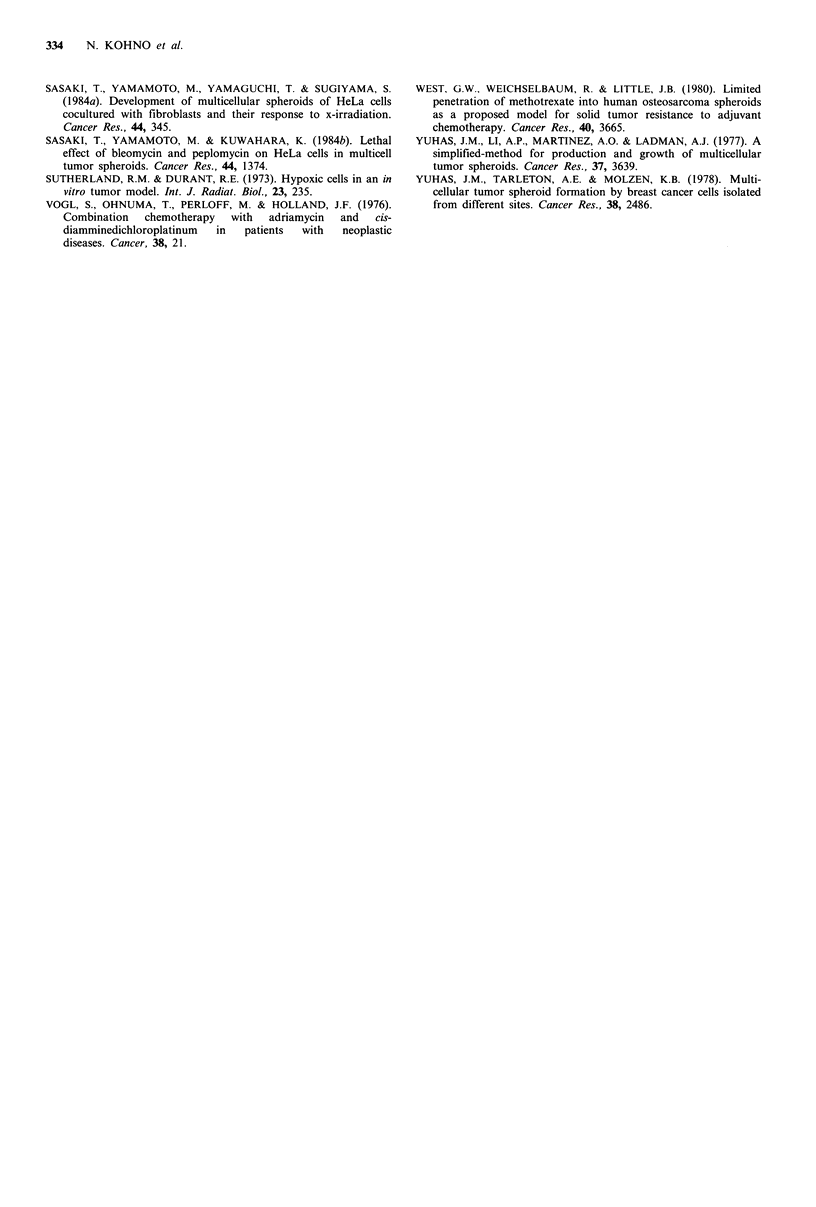# Interactions of doxorubicin and cis-platin in squamous carcinoma cells in culture.

**DOI:** 10.1038/bjc.1988.213

**Published:** 1988-09

**Authors:** N. Kohno, T. Ohnuma, M. Kaneko, J. F. Holland

**Affiliations:** Department of Neoplastic Diseases, Mount Sinai School of Medicine, New York, NY 10029.

## Abstract

**Images:**


Br. J. Cancer (1988), **58**, 330–334 © The Macmillan Press Ltd., 1988

### Interactions of doxorubicin and *cis*-platin in squamous carcinoma cells in culture

N. Kohno, T. Ohnuma, M. Kaneko & J.F. Holland

*Departments of Neoplastic Diseases, Pathology and the Derald H. Ruttenberg Cancer Center, Mount Sinai School of Medicine, 1 Gustave L. Levy Place, New York, NY10029, USA.*

**Summary** Doxorubicin (DXR) has a positive inoculum effect and penetrates poorly into the core of multicellular tumour spheroids (MTS). *Cis*-platin (DDP) displays neither of these characteristics. We evaluated whether combining these 2 agents would influence the cell kill effect at a tumour mass level. MTS were produced from a PC-10 squamous lung carcinoma cell line. MTS were exposed to either drug first for 1 h with different intervals between exposure. Cells were then trypsinized to a single cell suspension and subjected to clonogenic assay. Combination effects were analyzed by median effect plot analysis. The more MTS $ml^{-1}$ medium, the lower the cell kill effect of DXR. Simultaneous exposure to the 2 drugs was synergistic. DXR exposure first followed by DDP was less efficacious than, or the same as, the simultaneous exposure. In contrast, DDP followed by DXR was more efficacious with the best cell kill at a 1 h interval between each drug. This phenomenon was observed even at non-toxic doses of DDP. The fluorescent microscopic study of DXR indicated that prior exposure of MTS to DDP resulted in increased DXR penetration into the MTS core leading to heightened synergism with this sequence. These data suggest that the proper combination of DXR plus DDP should be in sequence with DDP first. Clinical, toxicological and pharmacological trials of DDP administration first, followed by DXR, are warranted.

Doxorubicin (DXR) and *cis*-platin (DDP) are both highly active anticancer agents widely used in the treatment of human cancer. We introduced a combination of these 2 agents, termed A & P (adriamycin and platinum) (Vogl *et al.*, 1976), which has become the basis, by adding more drugs, for the treatment of patients with ovarian cancer, small cell lung cancer and other neoplasms. Recently, we have demonstrated that DXR has a positive inoculum effect *in vitro* and loses efficacy at high cell densities, whereas DDP does not (Ohnuma *et al.*, 1986). Moreover, we have shown that DXR penetrates poorly into the core of multicellular tumour spheroids (MTS) whereas DDP shows no such penetration gradient (Inoue *et al.*, 1985). These pharmacological differences at the tumour mass level indicate that DXR should be best used when tumour size is small and cell density low. Therefore, we hypothesized that the proper sequence of A & P should be DDP first, followed by DXR, rather than *vice versa*. This communication sets forth the experimental proof of our hypothesis and elucidates a possible mechanism for this effect.

### Materials and methods

### *Human tumour cell line*

PC-10 squamous lung carcinoma cell line was used in these experiments (Kinjo *et al.*, 1979). Cells were maintained as a monolayer in RPMI-1640 medium (GIBCO, Grand Island, NY), supplemented with 10% (v/v) heat-inactivated foetal bovine serum (FBS) (Gibco) at 37°C in a 5% $CO_2$/95% humidified air atmosphere. These cells were subcultured after trypsinization with 0.2% trypsin (Type III from bovine pancreas, Sigma, St Louis, MD) and 0.01% EDTA in Hank's balanced salt solution (HBSS) (Gibco).

### *Growth of MTS*

MTS were developed by a liquid overlay culture technique (Yuhas *et al.*, 1977), as described previously (Kohno *et al.*, 1987). Aliquots of $1 \times 10^5$ cells in 10 ml of complete culture medium were placed in 100 mm plastic Petri dishes (Falcon 1005, Cockeysville, MD) previously coated with 0.5% agar (Noble, Difco, Detroit, MI) in the same culture medium. These cells were incubated in 5% $CO_2$/95% humidified air at 37°C. When MTS were formed, they were transferred to a new agar dish once a week.

### *Drugs*

DXR was purchased from Adria Laboratories, Columbus, OH and DDP was purchased from Bristol Laboratories, Syracuse, NY.

### *Conditions of drug exposure and determination of cell survival*

After 3 weeks of culture, MTS with a diameter of $\sim 700\,\mu m$ were formed. MTS with a diameter $> 700\,\mu m$ tended to develop a necrotic core. MTS with $\sim 700\,\mu m$ were transferred into a new agar-coated multiwell plate (Falcon 3046, Becton Dickinson Labware, Lincoln Park, NJ) containing 2 ml fresh medium. After a preincubation period of 24 h, MTS in different densities were exposed to graded concentrations of DXR or DDP for 1 h. The MTS were then gently washed twice with PBS (Gibco). The single cell suspensions were made by exposure to the 0.2% trypsin and 0.01% EDTA solution for 10 min at 37°C, followed by mechanical disaggregation through repeated pipetting. These cells were washed once with the medium and resuspended in the medium. One tenth ml aliquots of cell suspension (3,000 cells) were seeded on 60 mm Petri dishes (Corning 25011, Corning, NY) containing 0.5% noble agar in complete culture medium for clonogenic assay (Kuroki, 1974). The dishes were incubated for 10 days at 37°C, under 5% $CO_2$-95% humidified air. Colonies of $\geq 50$ cells were counted. The plating efficiency of untreated cells under these conditions was $\sim 40\%$. The dose response curve was drawn by plotting the number of colonies as a percentage of control against each drug concentration. Each experiment was done in triplicate and repeated at least 3 times.

Combination experiments were carried out as a 1 h exposure to each drug. After drug exposure, cells were washed free of drug and incubated in drug-free culture medium for different intervals. After the indicated incubation time periods, the MTS were exposed to a second drug for 1 h, washed free of drug. trypsinized to a single cell suspension and subjected to clonogenic assay.

Correspondence: T. Ohnuma.
Received 21 January 1988; and in revised form, 19 May 1988.

*Data analysis*

The efficacy of the combination was determined by the median effect plot analysis using an IBM PC microcomputer system (Chou & Talalay, 1984, 1987; Chou, 1985). This method involves plotting dose-effect curves for each drug for one or more multiple-dilutions and fixed ratio combinations of the drugs using the median effect equation: $fa/fu = (D/D_m)^m$, where $D$ is the dose, $D_m$ is the dose required for 50% effect (e.g. 50% inhibition of PC-10 cell's colony formation), $fa$ is the fraction affected by the dose $D$, $fu$ the fraction unaffected and $m$ a coefficient signifying the sigmoidicity of the dose-effect curve. The dose-effect curve was plotted using a logarithmic conversion of this equation which determines the $m$ and $D_m$ values. Based on the slope of the dose-effect curves, it can be decided whether the agents have mutually exclusive effects (e.g. similar mode of action) or mutually non-exclusive effects (e.g. independent mode of action). A combination index (CI) was then determined using the equation:

$$CI = \frac{(D)_1}{(Dx)_1} + \frac{(D)_2}{(Dx)_2} + \frac{\alpha(D)_1(D)_2}{(Dx)_1(Dx)_2}$$

where $(Dx)_1$ and $(Dx)_2$ are the doses of drugs 1 and 2, respectively, required to produce x% effect individually. $(Dx)_1$ and $(Dx)_2$ for x% cell kill can be determined by drawing a least square regression line on the computer graphic system. $(D)_1$ and $(D)_2$ are the doses of drugs 1 and 2, respectively, which are required to produce the same x% effect in combination. If the drugs are mutually exclusive, then $\alpha$ is 0: if mutually non-exclusive, then $\alpha$ is 1. When $CI = 1$, the interaction was considered additive; when $CI < 1$, synergism was indicated; and when $CI > 1$, antagonism was indicated.

*Fluorescent microscopy*

MTS of ~700 μm diam. were treated with $2\times10^{-5}$ M of DXR alone or combination of DXR and $2\times10^{-5}$ M DDP at 1 h intervals of each drug, which was the best cell kill sequence. The treated MTS were washed with ice-cold PBS once, embedded in OCT compound (Miles Scientific, IL) and frozen rapidly. Thin sections (5 μm) were made using a cryotome (Lipshaw Elect, MI). DXR fluorescence was observed under a fluorescent microscope equipped with epi-illumination (Nikon, DS-EPI-FL, Japan) using G-Green (excitation filter 535–550 μm, barrier filter 580 μm). In this system endogenous fluorescence from control MTS (ones not exposed to DXR) could not be recognized.

### Results

The influence of MTS density on DXR- or DDP-induced cell lethality is shown in Figure 1. For DXR-induced cell lethality, the higher the density of MTS, the lesser the cell kill effects and dose-response curves of DXR progressively flattened at high drug concentrations. In contrast, DDP gave entirely different dose-effect curves; cell survival curves for high MTS density and low density overlapped each other and there was progressively increasing cell kill at increasing doses.

Time dependent cell lethality of the combination of DXR plus DDP at low MTS density is illustrated in Figure 2. Three lines are shown for 3 different concentrations of the drug. DXR exposure followed by DDP was less efficacious than, or the same as, simultaneous exposure. In contrast, exposure to DDP followed by DXR was more efficacious; the best cell kill with a 1 h interval between each drug. Increasing time intervals from DDP to DXR for longer than 1 h resulted in a gradual diminution of the potentiation.

The influence of MTS density on time-dependent cell lethality of DXR plus DDP combination is shown in Figure

**Figure 1** Influence of the density of PC-10 squamous carcinoma multicellular tumor spheriods on doxorubicin (panel a) or *cis*-platin (panel b) – induced cell lethality. Bar, s.d.

**Figure 2** Time-dependent cell lethality of doxorubicin plus *cis*-platin on multicellular tumour spheriods. Three different concentrations of doxorubicin and *cis*-platin tested are shown on the right side of each line. Bar, s.d.

3. The differences of the 2 lines on the left half of the panel indicate weak activity of the combination at high MTS density when DXR is administered first. When the 2 drugs were given simultaneously, the survival fractions were nearly identical, indicative of better expression of DDP activity. For both low and high MTS densities, the best cell kill sequence of this combination was DDP followed by DXR with a 1 h interval between the 2 drugs. When the interval between DDP followed by DXR widened, the 2 lines began to separate again, suggesting the same effect of MTS density seen when DXR was given first.

Examples of the median effect plot analysis from the combination – DXR followed 24 h later by DDP, simultaneous exposure of the 2 drugs and DDP followed 1 h later by DXR – are shown in Figure 4. All of these 3 lines showed synergistic interactions; among them, the DDP expo-

**Figure 3** Influence of the density of multicellular tumour spheroids on time-dependent cell lethality of doxorubicin plus *cis*-platin. The line for the low density of multicellular tumour spheroids (2 MTS ml$^{-1}$) was taken from **Figure 2**. Drug concentrations used were doxorubicin $2 \times 10^{-5}$ M and *cis*-platin $2 \times 10^{-5}$ M. Bar, s.d.

**Figure 4** Time-dependent combination effects of doxorubicin plus *cis*-platin against multicellular tumour spheroids expressed as combination index.

sure, followed 1 h later by DXR, was the most efficacious sequence.

Pretreatment with low concentrations of DDP, which was non-toxic to cells in monolayer, also produced increased DXR-induced cell kill: $1 \times 10^{-7}$ M DDP produced a decrease in surviving fraction up to 30% for cells in MTS (Table I). These results indicate that DXR penetration was enhanced by non-toxic concentrations of *cis*-platin and that the penetration-enhancing effects can be separable from the cytotoxic effects of the compound.

Fluorescent microscopic observations of DXR penetration into MTS are illustrated in Figure 5. When MTS were

**Table I** Influence of the pretreatment with different concentrations of *cis*-platin on doxorubicin-induced cell lethality. Data are shown as survival fraction in percentage compared to doxorubicin alone control ($\pm$s.d.)

| *Cis-platin (M)* | *Monolayer* | *Multicellular tumour spheroids* |
|---|---|---|
| 0 | 100.0[a] | 100.0[b] |
| $1 \times 10^{-9}$ | $103.4 \pm 0.9$ | ND |
| $1 \times 10^{-8}$ | $103.4 \pm 1.5$ | $86.0 \pm 7.8$ |
| $1 \times 10^{-7}$ | $92.9 \pm 6.5$ | $71.7^{*} \pm 3.8$ |
| $1 \times 10^{-6}$ | $84.9 \pm 7.7$ | $63.7^{*} \pm 9.6$ |

[a]$1 \times 10^{-6}$ M doxorubicin alone; [b]$2 \times 10^{-5}$ M doxorubicin alone; *The asterisks indicate significant ($P < 0.05$) decrease in survival fraction as compared to doxorubicin alone control; ND, not done.

**Figure 5** Panel a shows doxorubicin fluorescence of spheroid cross-section after 1 h exposure to the compound alone. Panel b shows doxorubicin fluorescence of spheroid cross-section after pre-treatment with *cis*-platin.

exposed to DXR alone, DXR fluorescence was seen in only a few outerlayer(s) of the MTS. When MTS were exposed to DDP 1 h previously – the best cell kill sequence – DXR fluorescence was seen within the entire layers of the MTS (Figure 5, Panel b).

### Discussion

MTS have certain characteristics similar to *de novo* solid tumours (Allison *et al.*, 1983; Franko & Sutherland, 1979; Freyer & Sutherland, 1980; Nederman *et al.*, 1984; Sutherland & Durant, 1973; Yuhas *et al.*, 1977, 1978). They contain such extracellular matrix as fibronectin, laminin and collagen (Nederman & Twentyman, 1984), comprise a chronically hypoxic cell population in the core (Franko & Sutherland, 1979; Sutherland & Durant, 1973) and show heterogenous cell cycle times (Allison *et al.*, 1983; Freyer & Sutherland, 1980). For these reasons, MTS have been used as an *in vitro* model to study the effects of radiation (Allison *et al.*, 1983; Franko & Sutherland, 1979; Freyer & Sutherland, 1980; Sasaki *et al.*, 1984*a*; Sutherland & Durant, 1973) and chemotherapeutic agents (Erlichman & Vidgen, 1984; Kerr *et al.*, 1986; Nederman & Twentyman, 1984; Sasaki *et al.*, 1984*b*; West *et al.*, 1980).

Our data show that the density of MTS influences the cell kill effect of certain drugs. The higher the density of MTS, the lesser the cell kill effect of DXR. The concept of inoculum effect seen at a single cell level could thus be extended to the density of MTS. Since solid tumour masses *in vitro* do not grow more than 300–400 μm in diameter without a blood supply (Folkman, 1986; Kolstad, 1968), we have assumed that a large clinically recognizable tumour mass with neovasculature is equivalent to MTS in high density. The DDP-induced cell kill effect seemed to follow first order kinetics irrespective of MTS density, indicating good drug penetration into the MTS core. In contrast, the cell kill effect of DXR was progressively less efficacious at higher drug concentrations as a consequence of poor drug penetration.

The combination study herein presented shows that sequencing DDP and DXR influenced the cell kill effect at a tumour mass level. As seen in Figure 4, DDP plus DXR was always synergistic, with the exception of very low concentrations of the drugs. With increasing effect levels the combination index was progressively lower indicating heightened synergism. The drug sequencing studies showed that the best cell kill sequence was DDP first with a 1 h interval before DXR exposure. The increased synergistic interaction of this sequence was shown to be due to an increased population of cells in MTS at risk.

In attempts to elucidate the mechanism of the heightened cell kill effect from this sequence, we evaluated whether DDP could influence cell lethality and penetration of DXR. Pretreatment of DDP increased DXR-induced cell lethality even at non-toxic low concentration levels for MTS. This was not observed for monolayers. For fluorescent microscopy study, DXR concentrations of $\geqq 2 \times 10^{-5}$ M were necessary in order to detect the fluorescence. Exposure to DXR alone resulted in DXR fluorescence only on one or two outer layer(s) of the MTS (Inoue *et al.*, 1985). When MTS were exposed to DDP and DXR in this sequence, DXR fluorescence was seen throughout the MTS, indicating good penetration of DXR. The precise mechanism of DDP-induced improvement in DXR penetration is unclear. Other workers indicated that drug penetration into MTS is influenced by (a) cell to cell interaction in MTS, (b) molecular size of the drug, (c) liquid solubility of the drug and (d) intraspheroidal pH gradients (Erlichman & Vidgen, 1984; Kerr *et al.*, 1986). It is likely that DDP exposure changed cell-to-cell interactions within the MTS, making it easier for DXR to penetrate into the core. While the interaction between DDP and cell membrane is poorly understood, it has been observed that the compound binds with cell surface DNA, leading to the loss of the nucleic acid (Juckett & Rosenberg, 1982). Erlichman and Vidgen (1984) reported that avid binding of DXR to the outer layer of MTS inhibited penetration of the drug into the core. It is possible that the avid binding of DXR to cell surface material such as DNA was inhibited by pretreatment with DDP.

Theoretical and practical aspects of combination chemotherapy at a single cell level have been discussed by Sartorelli and Creasey (1982). Little information has so far been provided, however, on the approach with combination chemotherapy at the tumour mass level. Sensitivity of a cell to DXR and DDP is known to be highly related to the intracellular drug concentration (Eichholtz-Wirth & Hietel, 1986; Iliakis & Lazor, 1987). Any means to increase the drug penetration into MTS should improve drug concentration within the cells in the MTS core.

Our observations indicate that initial treatment with DDP resulted in increased efficacy for DXR at the tumour mass level. Clinical, toxicological and pharmacological trials of DDP followed by DXR are warranted.

This work was supported in part by United States Public Health Service grants CA-15936 from the National Cancer Institute, Bethesda, MD; by the Iwama Memorial Sony International Fellowship, New York, NY; by the Chemotherapy Foundation, Inc., New York, NY and by the T.J. Martell Foundation for Leukemia and Cancer Research, New York, NY. We thank Dr T.-C.. Chou for the median effect plot analysis of our laboratory data and Dr S.N. Thung for the fluorescent microscopic study.

### References

ALLISON, D.C., YUHAS, J.M., RIDOLPHO, P.F., ANDERSON, S.L. & JOHNSON, T.S. (1983). Cytophotometric measurement of the cellular DNA content of [$^3$H] thymidine-labelled spheroids. Demonstration that some non-labelled cells have S and $G_2$ DNA content. *Cell Tissue Kinet.*, **16**, 237.

CHOU, T.C. & TALALAY, P. (1984). Quantitative analysis of dose-effect relationships: The combined effects of multiple drug or enzyme inhibitors. *Advances in Enzyme Regulation*, **22**, 27.

CHOU, T.C. (1985). New computerized quantitative approach to combination therapy. *Proc. Am. Assoc. Cancer Res.*, **26**, 341. (*abstract*).

CHOU, T.C. & TALALAY, P. (1987). Applications of the median-effect principle for the assessment of low-dose risk of carcinogens and for the quantitation of synergism and antagonism of chemotherapeutic agents. In *New Avenues in Developmental Cancer Chemotherapy*, Harpar, R. and Connors, A. (eds) p. 37. Academic Press, New York.

EICHHOLTZ-WIRTH, H. & HIETEL, B. (1986). The relationship between *cis*-platin sensitivity and drug uptake into mammalian cells *in vitro*. *Br. J. Cancer*, **54**, 239.

ERLICHMAN, C. & VIDGEN, D. (1984). Cytotoxicity of adriamycin in MGH-U1 cells grown as monolayer cultures, spheroids and xenografts in immune-deprived mice. *Cancer Res.*, **44**, 5369.

FOLKMAN, J. (1986). How is blood vessel growth regulated in normal and neoplastic tissue? G.H.A. Clowes Memorial Award Lecture, *Cancer Res.*, **46**, 467.

FRANKO, A.J. & SUTHERLAND, R.M. (1979). Radiation survival of cells from spheroids grown in different oxygen concentrations. *Radiat. Res.*, **79**, 454.

FREYER, J.P. & SUTHERLAND, R.M. (1980). Selective dissociation and characterization from diffferent regions of multicell tumor spheroids. *Cancer Res.*, **40**, 3956.

ILIAKIS, G. & LAZOR, W. (1987). Reduction by caffeine of adriamycin-induced cell killing and DNA damage in chinese hamster cells: Correlation with modulation in intracellular adriamycin content. *Cancer Res.*, **47**, 2224.

INONE, S., OHNUMA, T., HOLLAND, J.F. & WASSERMAN, L.R. (1985). Susceptibility of multicellular tumor spheroids (MTS) to doxorubicin (DXR) and *cis*-platin (DDP). *Proc. Am. Assoc. Cancer Res.*, **26**, 341. (*abstract*).

JUCKETT, D.A. & ROSENBERG, B. (1982). Action of *cis*-diamminedichloro-platinum on cell surface nucleic acids in cancer cells as determined by cell electrophoresis techniques. *Cancer Res.*, **42**, 3565.

KERR, D.J., WHELDON, T.E., KERR, A.M., FRESHNEY, R.I. & KAYE, S.B. (1986). The effect of adriamycin and 4′-deoxydoxorubicin on cell survival of human lung tumor cells grown in monolayer and as spheroids. *Br. J. Cancer*, **54**, 423.

KINJO, M., OKA, K., NAITO, S. & 5 others (1979). Thromboplastic and fibrinolytic activities of cultured human cancer cell lines. *Br. J. Cancer*, **39**, 15.

KOHNO, N., OHNUMA, T., HOLLAND, J.F. & BILLER, H. (1987). Effect of anti-cancer agents on the shedding of cells from human multicellular tumor spheroids. *Invasion Metast.*, **7**, 264.

KOLSTAD, P. (1968). Intercapillary distance, oxygen tension and local recurrence in cervix cancer. *Scand. J. Clin. Lab. Invest. Suppl.*, **106**, 145.

KUROKI, T. (1974). Colony formation of mammalian cells on agar plates and its application to Lederberg's replica plating. *Exp. Cell Res.*, **80**, 55.

NEDERMAN, T., NORLING, B., GLIMELIUS, B., CARLSSON, J. & BRUNK, U. (1984). Demonstration of an extra-cellular matrix in multicellular tumor spheroids. *Cancer Res.*, **44**, 3090.

NEDERMAN, T. & TWENTYMAN, P. (1984). Spheroids for studies of drug effects. In *Recent Results in Cancer Research*, Acker *et al.* (eds) p. 84. Springer-Verlag–Berlin–Heidelberg.

OHNUMA, T., ARKIN, H. & HOLLAND, J.F. (1986). Effects of cell density on drug-induced cell kill kinetics *in vitro* (inoculum effect). *Br. J. Cancer*, **54**, 415.

SARTORELLI, A.C. & CREASEY, W.A. (1982). Combination chemotherapy. In *Cancer Medicine*, Holland, J.F. & Frei III, E. (eds) p. 720. Lea and Febiger: Philadelphia.

SASAKI, T., YAMAMOTO, M., YAMAGUCHI, T. & SUGIYAMA, S. (1984*a*). Development of multicellular spheroids of HeLa cells cocultured with fibroblasts and their response to x-irradiation. *Cancer Res.*, **44**, 345.

SASAKI, T., YAMAMOTO, M. & KUWAHARA, K. (1984*b*). Lethal effect of bleomycin and peplomycin on HeLa cells in multicell tumor spheroids. *Cancer Res.*, **44**, 1374.

SUTHERLAND, R.M. & DURANT, R.E. (1973). Hypoxic cells in an *in vitro* tumor model. *Int. J. Radiat. Biol.*, **23**, 235.

VOGL, S., OHNUMA, T., PERLOFF, M. & HOLLAND, J.F. (1976). Combination chemotherapy with adriamycin and *cis*-diamminedichloroplatinum in patients with neoplastic diseases. *Cancer*, **38**, 21.

WEST, G.W., WEICHSELBAUM, R. & LITTLE, J.B. (1980). Limited penetration of methotrexate into human osteosarcoma spheroids as a proposed model for solid tumor resistance to adjuvant chemotherapy. *Cancer Res.*, **40**, 3665.

YUHAS, J.M., LI, A.P., MARTINEZ, A.O. & LADMAN, A.J. (1977). A simplified-method for production and growth of multicellular tumor spheroids. *Cancer Res.*, **37**, 3639.

YUHAS, J.M., TARLETON, A.E. & MOLZEN, K.B. (1978). Multicellular tumor spheroid formation by breast cancer cells isolated from different sites. *Cancer Res.*, **38**, 2486.